# Polyurethanes Crosslinked with Poly(vinyl alcohol) as a Slowly-Degradable and Hydrophilic Materials of Potential Use in Regenerative Medicine

**DOI:** 10.3390/ma11030352

**Published:** 2018-02-27

**Authors:** Justyna Kucińska-Lipka

**Affiliations:** Department of Polymers Technology, Faculty of Chemistry, Gdansk University of Technology, Narutowicza St. 11/12, 80-233 Gdansk, Poland; juskucin@pg.gda.pl

**Keywords:** polyurethane, poly(vinyl alcohol), crosslinking, degradation, surface, hydrophilicity, bone tissue engineering

## Abstract

Novel, slowly-degradable and hydrophilic materials with proper mechanical properties and surface characteristics are in great demand within the biomedical field. In this paper, the design, synthesis, and characterization of polyurethanes (PUR) crosslinked with poly(vinyl alcohol) (PVA) as a new proposition for regenerative medicine is described. PVA-crosslinked PURs were synthesized by a two-step polymerization performed in a solvent (dimethylsulfoxide, DMSO). The raw materials used for the synthesis of PVA-crosslinked PURs were poly(ε-caprolactone) (PCL), 1,6-hexamethylene diisocyanate (HDI), and PVA as a crosslinking agent. The obtained materials were studied towards their physicochemical, mechanical, and biological performance. The tests revealed contact angle of the materials surface between 38–47° and tensile strength in the range of 41–52 MPa. Mechanical characteristics of the obtained PURs was close to the characteristics of native human bone such as the cortical bone (T_Sb_ = 51–151 MPa) or the cancellous bone (T_Sb_ = 10–20 MPa). The obtained PVA-crosslinked PURs did not show significant progress of degradation after 3 months of incubation in a phosphate-buffered saline (PBS). Accordingly, the obtained materials may behave similar to slowly-degradable materials, which can provide long-term physical support in, for example, tissue regeneration, as well as providing a uniform calcium deposition on the material surface, which may influence, for example, bone restoration. A performed short-term hemocompatibility study showed that obtained PVA-crosslinked PURs do not significantly influence blood components, and a cytotoxicity test performed with the use of MG 63 cell line revealed the great cytocompatibility of the obtained materials. According to the performed studies, such PVA-crosslinked PURs may be a suitable proposition for the field of tissue engineering in regenerative medicine.

## 1. Introduction

Polyurethanes (PURs) are characterized as biocompatible and hemocompatible materials. Thus, they are widely developed in the medical field [1]. PURs possess a segmented structure, which consists of hard and soft segments. Soft segments (SS) are formed by the macrodiol blocks, while hard segments (HS) are derived from diisocyanate and chain extenders [2]. According to the segmented structure of PURs, there is an easy way to adjust their mechanical properties to expected requirements. Due to this, PURs are used in many novel fields of medical sciences, such as regenerative medicine and tissue engineering (TE) [3,4]. 

The aim of TE is to assemble constructs that provide mechanical, cellular, and molecular signals to restore, maintain, or improve damaged tissues or whole organs. Therefore, bone tissue engineering strives to restore and heal bone in musculoskeletal disorders, injuries, or deformities. Nowadays, autografts and allografts are commonly used in the clinical practice of restorative therapy [1,4,5]. However, bone harvesting is traumatic, causes pain and infections at the donor site, and very often results in complications. Hence, the use of synthetic grafts is emerging as an alternative treatment [1,5].

One of the requirements of bone tissue engineering is that the materials must be biodegradable. The degradation of PURs may be controlled not only by the type of macrodiol, diisocyanate and chain extender chosen for the synthesis but also by the morphology, hard to soft segments ratio and the degree of crosslinking. Properly designed PURs are biodegradable [6,7] and they degrade mainly through hydrolytic degradation of the soft segment derived from macrodiols having ester moieties [8]. In physiological conditions, degradation of the urethane linkages (UL) is unlikely. This is because the degradation rate of UL is an order of magnitude lower than the degradation rate of ester linkages. Biodegradable PURs are mainly synthesized from macrodiols containing ester moieties i.e., poly(ε-caprolactone)diol (PCL), poly(glycolic acid) (PGA), poly(lactic acid) (PLA) and their application is FDA (Food and Drug Administration) approved [9,10].

The ideal bone graft promotes calcification of the newly formed tissue in vivo. The ability to support calcium phosphate crystal formation is one of the most unique properties that distinguishes PURs from other biomaterials and predisposes them to be used in bone regeneration. PURs, when they are implanted into the circulatory system, within connective tissue, undergo calcification [11,12,13]. It is presumed that the hydrophilicity and the presence of ether oxygen have the greatest impact on calcification [14]. In this case, hydrophobic polyester-urethanes calcification occurs only on the surface, which is in a direct contact with body fluids [15]. In case of hydrophilic poly(ether-uretane)s, it occurs both on the surface and within the polymer [16]. Moreover, PURs have a relatively high affinity to calcium phosphates [16,17,18]. 

Among crosslinked materials used in the biomedical field, the literature reports hydrogels obtained by using natural polymers such as gelatin [19], chitosan [19,20], hyaluronic acid [21], collagen [20], and synthetic once such as poly(vinyl alcohol) (PVA) [22] or poly(vinylpirolidone) (PVP) [23]. PVA is a useful polymer in the biomedical field. It is linear in its structure, which is highly soluble in water and resistant to most organic solvents. The FDA has approved PVA to be in close contact with food products and to be used as a medical device due to its biocompatibility, nontoxicity, non-carcinogenicity, swelling and bio-adhesive properties [22]. Until now, PVA hydrogels and membranes have been developed for biomedical applications such as contact lenses [24], artificial pancreases [25,26], hemodialysis [27], and synthetic vitreous humor [28], as well as for implantable medical materials to replace cartilage [29,30,31,32,33] and meniscus tissues [34,35]. It is an attractive material for these applications because of its biocompatibility and low protein adsorption properties, resulting in low cell adhesion compared with other hydrogels. These diverse uses of PVA in medical devices indicate that it is safe for human use in applications where adsorption of the host protein is undesired and the device experiences tensile stress during use [36].

The aim of performed studies was to design, synthesize and characterize PVA-crosslinked PURs as a proposition of the novel material, which can be utilized in the field of regenerative medicine. The data reported in this paper constituted the basis of the Polish patent No. PL 223226 B1 [37] released in 2016. Until now, only a few scientific papers have been related to the PVA combination with PUR for biomedical applications, and such systems are mainly crosslinked hydrogels. For example, Bonakdr et al. reported a PVA hydrogel crosslinked by biodegradable polyurethane for tissue engineering of cartilage. It was a PVA hydrogel crosslinked by urethane prepolymer consisting of cycloaliphatic 4,4’-bis cyclo(hexyl isocyanate) (HMDI) and PCL [38]. Studies by Shokrgozar et al. [39] confirmed that PVA hydrogels crosslinked by polyurethane chain are good candidates for a rabbit cartilage model. Petrini et al. [40] suggested the application of polyurethane hydrogels, obtained by using HMDI, PEG, low molecular chain extender 1,4-butandiol and two types of catalysts, 1,4-diazabicyclo [2.2.2]octane (DABCO) and dibutyltin dilaurate (DBTDL), for the biomedical field. The obtained hydrogels had a porous structure of low crosslinking degree, which allowed obtaining of high swelling and not water-soluble PVA-PUR hydrogels. These materials also present an elastomeric behavior in the swollen state [40]. 

The novel PVA-crosslinked PURs, described in this paper, were obtained in a two-step polymerization procedure with DMSO used as a solvent. The chemical composition and properties of obtained PVA-crosslinked PURs were determined and compared to non-crosslinked PUR samples. PUR chemical composition was studied by using Fourier transform infrared spectroscopy (FTIR). The mechanical properties such as tensile strength percent of elongation at break and hardness were determined. A long-term interaction study was performed to verify these materials’ potential for degradation under conditions that simulate the environment of the human body. Before and after this examination the surface contact angle was studied. Short-term hemocompatibility was studied to indicate whether obtained materials did not cause a severe effect on blood components and the cytotoxicity test with the use of MG 63 cell line (Human osteosarcoma) was performed to indicate the effect of obtained materials on cells in vitro. In summary, the obtained material may potentially be used as biomedical material in the field of regenerative medicine.

## 2. Experimental

### 2.1. Materials and Methods

#### 2.1.1. Synthesis of Polyurethanes (PURs)

PURs were synthesized by the standard two-step polymerization procedure [3,4,36], which was carried out in DMSO solvent. Cast PURs, which were non-crosslinked (PU-I), were synthesized as follows: The urethane prepolymer (8 wt % of free isocyanate groups) was obtained in the reaction of poly(ε-caprolactone) (PCL, CAPA 2000, POCH, Gliwice, Poland), and aliphatic 1,6-hexamethylene diisocyanate (HDI, Sigma Aldrich, Poznań, Poland) dissolved in DMSO (30 wt % per the weight of the macrodiol used). The prepolymerization reaction was carried out at 90 °C for 5 h. In the second step the chain extender—1,4-butanediol (BDO) (POCH, Gliwice, Poland)—was added to the urethane prepolymer (after cooling up to 60 °C) to obtain PURs with a molar ratio of free isocyanate groups (NCO) (in the urethane prepolymer) to hydroxyl groups (OH) of chain extender BDO equal to NCO:OH = 0.95:1. Then, samples were transferred to the laboratory drier set at 100 °C for 24 h to complete the reaction.

**Figure 1:**
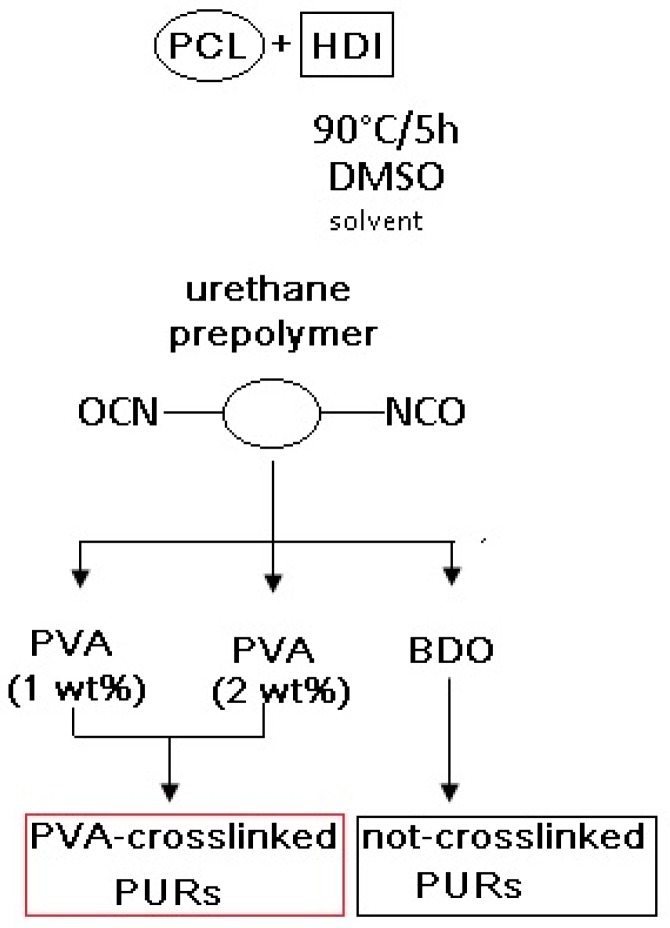
Scheme of PUR synthesis.

PURs crosslinked with poly(vinylalcohol) (PVA, Mw = 31,000, Mowiol 4-88, Sigma Aldrich, Poznań, Poland) (Table 1) were synthesized as follows: to the prepolymer (60 °C) different amounts (1 wt % or 2 wt % per weight of diisocyanate) of PVA were added and mixed for 2h to reach the solid material. Then, the samples were transferred to the laboratory drier set at 100 °C for 24 h to complete the reaction. The scheme of the reaction is presented in Figure 1. Symbols of the samples with their detailed composition are given in Table 1.

#### 2.1.2. Fourier Transform Infrared Spectroscopy (FTIR)

The FTIR analysis was performed with the use of a Nicolet 8700 Spectrometer (Thermo Fisher Scientific, Waltham, MA, USA) in the spectral range of 4000 to 500 cm^−1^ averaging 256 scans with a resolution of 4 cm^−1^. Spectra were analyzed by free software *Essential FTIR® Spectroscopy Toolbox 3.*

#### 2.1.3. Swelling and Crosslink Density

To perform sorption studies, six cylindrical samples (V = 3.5 cm^3^) were cut from the PU-I, PU-II-1 and PU-II-2, by using a sharp steel die. Samples (dried at 60 °C, RADWAG MAX50/SX, Radwag, Radom, Poland) were placed in glass containers and immersed in 15 mL of the organic solvents: DMSO. At regular time intervals the samples were taken out of the containers and the wet surfaces were gently pressed to remove surface-adsorbed solvents. The samples were weighed quickly and reimmersed in the respective solvents. The process was repeated until equilibrium was attained. The possibility of an error introduced due to the evaporation of solvent while weighing was minimized by weighing as quickly as possible within 30 s and taking the mean of 6 measurements. The sorption was carried out at 27 °C. Sorption (*S*) was calculated according to formula (1) where *m_i_*—sample weight after 120 h of incubation (g), *m*_0_—sample weight before the test (g) [41].

(1) $$S=\left(\frac{m_{i}-m_{0}}{m_{0}}\right)\cdot 100\%$$

The crosslink density study was performed according to the most common approach via a Flory-Rehner swelling experiment [41,42,43]. The crosslink densities of the samples (*ν*) were determined from measurements in a DMSO, using the Flory-Rehner relationship from the following Equation (2):

(2) $$\nu =\frac{-\ln\left(1-V_{r}\right)-V_{r}-\chi \cdot V_{r}^{2}}{V_{s}\cdot \left(V_{r}^{\frac{1}{3}}-\frac{1}{2}\cdot V_{t}\right)}=\frac{1}{Mc}$$

where, *Vs* is the molar volume of DMSO (*Vs* = 71 cm^3^/mol), *Vr* is the volume fraction of polymer in the sample at equilibrium swelling, *χ* is the Flory-Huggins natural rubber-DMSO interaction constant (*χ* = 0.38). The average molecular weight *Mc* (g/mol) of the polymer between crosslinks was calculated as an inverse of cross-link density (*ν*).

#### 2.1.4. Static Contact Angle (CA)

Static CA of PURs was determined at room temperature with the use of a Reichert Wien optical microscope (35× magnification, New York Microscope Company INC., Hicksville, New York, NY, USA). PURs were cut in 2 cm^2^ samples, whose surfaces were purified with n-hexane (POCH, Gliwice, Poland) before measurement. To determine contact angle the Sessile Drop Method (SDM) was applied, using a 5 µL of distilled water droplet. For each angle reported, at least ten measurements on different surface locations were averaged. The width and the height of the Sessile Drop were indicated, and the CA was determined according to the Formula (3)–(5).

(3) $$tg\theta =\frac{h}{d}$$

(4) $$\theta =arctg\frac{h}{d}$$

(5) $$\theta [{}^{\circ}]=\theta [rad]\cdot \frac{180{}^{\circ}}{\pi }$$

#### 2.1.5. Mechanical Properties

##### Tensile Strength (T_Sb_) and Elongation at Break (ε_b_) 

T_Sb_ and ɛ_b_ were studied by using the universal testing machine Zwick & Roell Z020 (Zwick Roell Polska Sp. Z o.o., Wrocław, Poland) according to PN-EN ISO 527-2:2012 [44] with a crosshead speed of 300 mm/min. Results were presented as an average of 6 measurements. 

##### Hardness

Hardness was measured by using the shore method according to [45]. The obtained data were presented with the shore degree (°Sh D and °Sh A). Ten measurements (each side of the PUR) were performed and results are an arithmetic mean of 10.

#### 2.1.6. Long-Term Interactions with Selected Media

The long-term interactions of obtained PURs were studied in selected media: canola oil, distilled water, and phosphate buffered saline (PBS) by the standard procedure [3,4]. PURs were cut into 6 round samples of V = 3.5 cm^3^. Prepared samples were dried and weighed in a thermobalance set at 60 °C. Then, 6 samples of each studied PVA-crosslinked PUR material were placed in a 24-well cell culture plate filled with canola oil, distilled water, or PBS. Canola oil is often used because it replaces the assay of lipids present in the living body. Furthermore, drugs delivered to the body encapsulated in a biodegradable polymer are often introduced as a lipid emulsion. Samples were incubated in a specific media at room temperature. Both distilled water and PBS simulate the environment of the human body fluids in vitro. Thus, they are used as a model media to determine progress of degradation. Different time points for different media were chosen by suggestions given in the literature [10,46]. 

Samples were incubated in media at 37 °C. Changes in the weight of the samples were examined after 24 h for canola oil medium, after 1 and 14 days for distilled water and after 1 and 3 months for PBS. Samples’ weight change measurements were as follows: samples were taken out from the container and put into a paper sheet to reduce the medium excess. Then, the samples were placed in the thermobalance (set at 60 °C) where they were dried and weighed to a constant weight. Mass loss was calculated by formula (6) where *m_i_* is sample weight after i-days of incubation (g)*, m*_0_ is sample weight before the test (g). In performed study the medium pH was controlled for PBS medium after 1 and 3 months by Metler Toledo pH-meter (Metler Toledo, Greifensee, Switzerland).

(6) $$S=\left(\frac{m_{i}-m_{0}}{m_{0}}\right)\cdot 100\%$$

#### 2.1.7. Optical Microscopy

The initial changes at the PURs′ surfaces were monitored by optical microscopy (OM, Bresser GmbH, Rhede, Germany) performed with the use of a Bresser microscope (Bresser GmbH, Rhede, Germany) at the magnification of 20×.

#### 2.1.8. Calcification Study

Golomb and Wagner’s Compound was used to perform the calcification study. The calcification metastable solution consisted of 3.87 millimole (mM) CaCl_2_, 2.32 mM K_2_HPO_4_, yielding a ratio of calcium to phosphate (Ca/PO_4_) = 1.67, and 0.05 M Tris Buffer (in this study C_4_H_11_NO_3_) dissolved in 1 mL of reverse osmosis (RO) water [25]. PUR and PUR-M samples were cut into round samples of 0.5 cm^2^ area. Prepared samples were dried and weighed in a thermobalance set at 60 °C. Then, 6 samples of each studied PUR material were placed in a 24-well cell culture plate filled with Golomb and Wagner’s Compound. The progress of the calcification was studied by Scanning Electron Microscopy (SEM, Zeiss Scanning Electron Microscope EV-40, Jena, Germany) with Energy Dispersive X-ray Spectroscopy (EDX) (Microanalyzer, Jena, Germany) after 21 days [47].

#### 2.1.9. Hemocompatibility 

Hemocompatibility was studied to evaluate the short-term action of obtained PVA-crosslinked PURs on blood components. Hemocompatibility was examined in a Medical Laboratory with analyzer SYSMEX XS–1000i (Symex Poland, Warszawa, Poland). Samples of venous blood from healthy women were used in this study. Biologic material, directly after being taken, was put into a test-tube containing heparin, an agent which prevents blood clotting. The next step was obtaining reference parameters for blood morphology. After that, in the test-tube were put samples with a size of 3.5 cm^3^ PU and 8 mL of blood was added. The samples before hemocompatibility test were sterilized with argon gas plasma generated over H_2_O_2_. The samples were incubated in blood for 15 and 240 min at room temperature. After this, they were removed and blood was hematologically analyzed.

The hemocompatibility test was performed according to Polish standards [48] (PN-EN ISO 15189), where no approval of an ethics committee is needed. It was performed at a certified laboratory in Gdansk Clinical Centre. The in vitro studies were performed using established MG 63 cell line according to the ISO Standard: ISO-10993-5:2009 [49].

#### 2.1.10. Biocompatibility

The biocompatibility studies were performed similarly to the cytotoxicity protocol given in our previous paper [47] as follows: 

##### In Vitro Cytocompatibility

The cytotoxicity assay was performed on the obtained materials: PU-I, PU-II-1 and PU-II-2. To examine the cytotoxicity of the samples, the extracts were prepared and tested on MG 63 cell line according to [49]. To obtain extract, the sterile samples were incubated in cell culture medium (Dulbecco’s Modified Eagle’s Medium, DMEM) (Gibco) supplemented with 10% fetal bovine serum (FBS) (Gibco), L-glutamine (1% solution in medium) (Gibco), 1% antibiotic–antimycotic mixture for 24 h at 37 °C under continuous steering. MG 63 cells were cultured in standard conditions (5% CO_2_, 37 °C, 95% humidity) in the culture polystyrene plates. MG 63 morphology was assessed (Nikon, Tokyo, Japan). Cells seeded without polymer extract in the culture medium served as the negative control. The viability of MG 63 has been investigated with 3-(4,5-dimethylthiazol-2-yl)-2,5-diphenyl tetrazolium bromide (MTT) test after 24 and 48 h of culture. The MTT test is a colorimetric assay routinely used in toxicology in vitro. It is based on the capacity of metabolically active cells to convert the substrate of the reaction, the yellow tetrazolium salt MTT 3-(4,5-dimethylthiazol-2-yl)-2,5-diphenyltetrazolium bromide, into the product, an insoluble formazan. The final product of the reaction was measured with an ELISA reader vs. Spectrophotometer (Molecular Devices LLC, San Jose, CA, USA) at 450 nm. MTT solution incubated without cells was used as blank and the signal was normalized to positive control (MG 63 cells cultured in standard plate). The viability rate (%) was expressed as a percentage of the positive control, where the severe cytotoxicity is observed for less than 30%, moderate cytotoxicity between 30–60%, slight cytotoxicity between 60–90%, and nontoxicity greater than 90% of viable cells [32].

The statistical analysis was performed with the use of the Origin Pro 8.5. To evaluate statistical differences the two-way method ANOVA (α = 0.05) and post hoc Tukey test (α = 0.05) were used.

##### Cell Adhesion 

Cell adhesion was investigated on the synthesized materials. Samples were placed in 24-well tissue culture polystyrene plates. Approximately 1 × 10^5^ MG 63 cells per well were seeded by dropping a cell suspension onto the surface of the examined materials. Cells seeded into wells without polymer samples served as a control. The cells were incubated in the same conditions as in the cytotoxicity assay. Cell attachment and distribution on the samples, after 24 and 72 h of incubation, were examined by staining with 4′,6-diamidine-2′-phenylindole dihydrochloride (DAPI) and Tetramethylrhodamine isothiocyanate mixed isomers (TRITC)-conjugated phalloidin for the immunofluorescent staining of cell nucleus and actin filaments in the cytoskeleton (Chemicon). The fluorescence observation of cell morphology was carried out with a Nikon Eclipse TE2000-U microscope (Precoptic, Warszawa, Poland).

## 3. Results

### 3.1. Fourier Transform Infrared Spectroscopy (FTIR)

**Figure 2:**
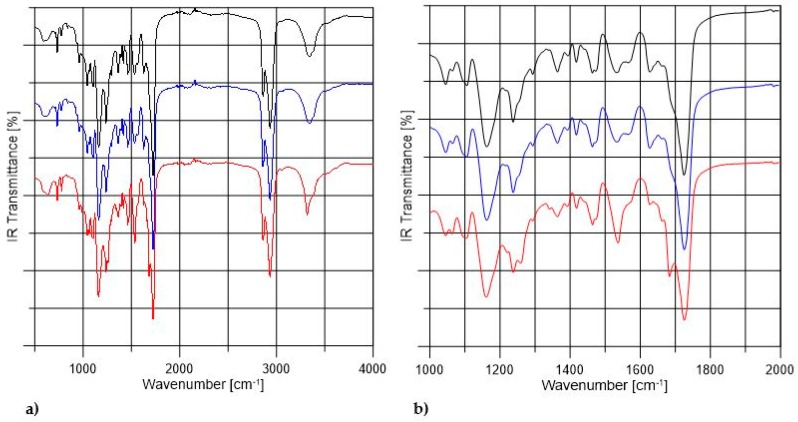
The FTIR spectra of non-crosslinked (black) and PVA-crosslinked PURs (blue and red respectively for 1 wt % and 2 wt % of PVA) in the range between 500–4000 cm^−1^ (**a**) and in the range of 1000–2000 cm^−1^ (**b**).

Figure 2 shows the FTIR spectra of non-crosslinked and PVA-crosslinked PURs. Table 2 shows the band assignments of obtained PUR samples. According to Figure 2 the composition of PUR materials was confirmed by bands assigned to the urethane linkages and their interactions in hard segments (HS). The complete reaction of used reagents was confirmed by the lack of free NCO band in the range between 2200–2300 cm^−1^. The wide stretching band of NH (3500 cm^−1^) confirmed formation of urethane bond in obtained materials. The stretching band of C=O was weaker by 20 cm^−1^ in the case of PU-II-1 and PU-II-2 than in the case of PU-I. Thus, the possible interactions between HS and OH of PVA might occur. The sharp band of C=O stretching (1730 cm^−1^) was related to the not-hydrogen-bonded C=O present in polyester PCL soft segments. On the other hand, the band noted at 1630 cm^−1^ was recognized as C=O, which was strongly hydrogen bonded, so engaged in the hydrogen bonds of HS in PUR structure. The strong hydrogen bonding between the HS present in the PUR structure was confirmed as well by the presence of bands related to the NH secondary amide deformations (1570–1540 cm^−1^) in urethane groups and stretching of CN in urethane linkage (1240 cm^−1^). Bands observed at 1160 cm^−1^, 1100 cm^−1^ and 1180 cm^−1^ were related to the combined asymmetric and symmetric stretching of -(C=O)-O-C- of ester and urethane groups. 

### 3.2. Swelling and Crosslink Density

Table 3 shows the behavior of the samples in DMSO, the Huggins parameter (*χ*) and crosslinking density (mol/cm^3^). Table 3 shows clearly that PU-I were soluble in DMSO, thus they were non-crosslinked. PURs obtained by PVA addition (PU-II-1 and PU-II-2) can be considered as crosslinked materials, because after the incubation they were swollen but not dissolved. Application of PVA of functionality >2 meant that some part of the OH groups, present in PVA, reacted with non-reacted NCO groups in the prepolymer and formed the crosslinks. According to the references the higher the crosslink density, the more degradation resistance the materials shows [50]. Thus, in this study, PURs with 2 wt % of PVA represented higher crosslink density than the PURs obtained by using 1 wt % of PVA and swelling of PU-II-2 is lower than PU-II-1.

### 3.3. Static Contact Angle Determination

Table 4 shows the CA of the surface of obtained PURs before and after 1 and 3 months of incubation in PBS. Table 4 shows that the addition of PVA to the PUR caused the obtaining of more hydrophilic materials (47 ± 0.1° and 38 ± 0.1° for PU-II-1 and PU-II-2 respectively) in comparison to the non-crosslinked PURs (59 ± 0.2°). On the other hand, the CA of obtained PURs did not significantly change after 3 months of incubation in PBS (Table 3). Thus, that may confirm that obtained materials were crosslinked [51,52]. 

### 3.4. Mechanical Properties

**Figure 3:**
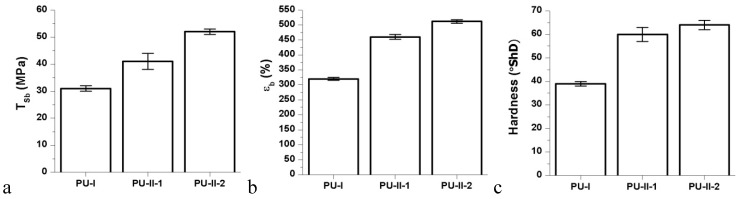
Tensile strength (T_Sb_) (**a**), percent of elongation at break (%) (ε_b_) (**b**); and hardness (**c**) of obtained non-crosslinked and PVA-crosslinked PURs.

Mechanical properties of obtained non-crosslinked and PVA-crosslinked PURs were presented in Figure 3. Figure 3 shows that each of the studied mechanical parameters (T_Sb_, ε_b_ and hardness) was increased due to the PVA-crosslinking of PURs. Thus, presence of crosslinks improves mechanical performance of the materials. Tensile strength of PVA-crosslinked PURs was 41 ± 3 MPa and 52 ± 1 MPa for PU-II-1 and PU-II-2 respectively, while for not-crosslinked PURs (PU-I) it was of 31 ± 1 MPa. In the case of percent of elongation at the break it was 460 ± 8% and 512 ± 6% respectively for PU-II-1 and PU-II-2, while for PU-I it was 320 ± 5%. Hardness for PU-II-1 and PU-II-2 was comparable (60 ± 3 °ShD and 64 ± 2 °ShD respectively) and higher than for PU-I (39 ± 1).

### 3.5. Long-Term Interactions with Selected Media

**Figure 4:**
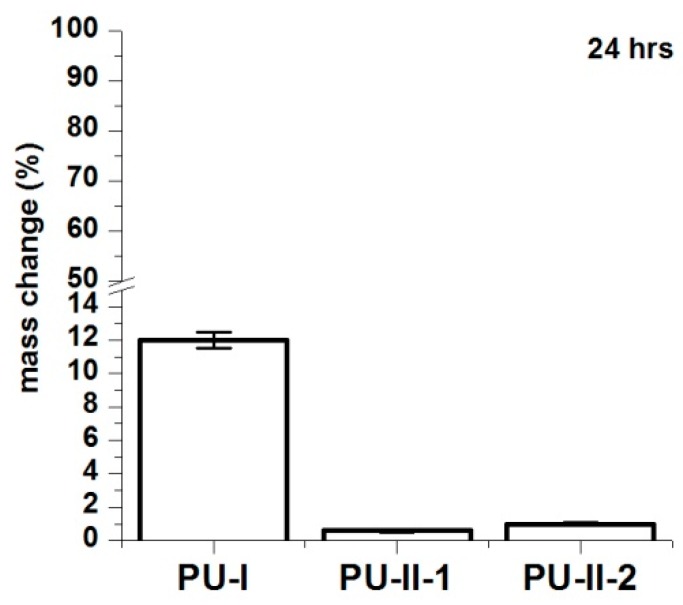
Canola oil sorption noted for PU-I, PU-II-1, PU-II-2 after 24 h of incubation.

**Figure 5:**
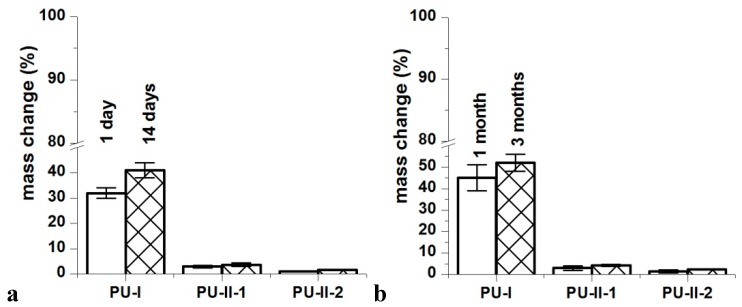
Distilled water (**a**) and PBS (**b**) sorption of obtained PURs after selected incubation time-points.

Figure 4 shows that the PVA-crosslinked PURs (PU-II-1 = 0.6 ± 0.1% and PU-II-2 = 1.0 ± 0.3%) had lower canola oil sorption capability in comparison to the linear and non-crosslinked PURs (PU-I = 12 ± 0.5%). Figure 5 shows that non-crosslinked PURs underwent defragmentation easily in distilled water (32 ± 2% and 41 ± 3% after 1 and 3 months of incubation respectively) in comparison to the PVA-crosslinked PURs, which stay stable during the incubation period (3 ± 0.5% and 3.7 ± 0.7% after one and 3 months of incubation respectively for PU-II-1; 1 ± 0.1% and 1.7 ± 0.1% after one and 3 months of incubation respectively for PU-II-2). In the case of the incubation in PBS medium the observed mass decrease was higher for non-crosslinked PURs (45 ± 6% after 1 month and 52 ± 4% after 3 months of incubation). The PVA-crosslinked PURs showed mass decrease (3 ± 1 % and 4.3 ± 0.5% respectively after one and 3 months of incubation for PU-II-1 and 1.5 ± 0.7% and 2.4 ± 0.2% after one and 3 months of incubation for PU-II-2) comparable to the one observed in case of distilled water. 

### 3.6. Optical Microscopy

**Figure 6:**
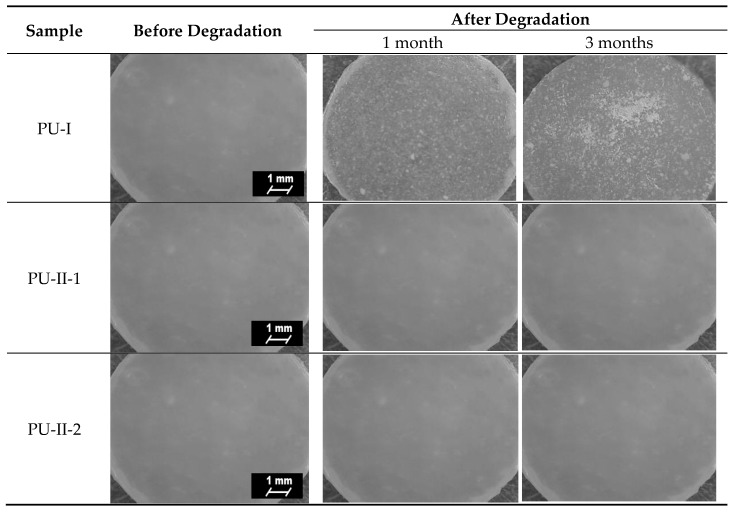
Microscopic images of the PUR surfaces before and after long-term interactions study performed in PBS.

Figure 6 presents microscopic images of obtained PURs before and after the incubation in PBS. Analysis of microscopic images of PVA-crosslinked PURs revealed no interruptions at their surface, which is in contrast to the non-crosslinked PURs. This suggests slow degradation of the material [53,54,55].

### 3.7. Calcification Study

**Figure 7:**
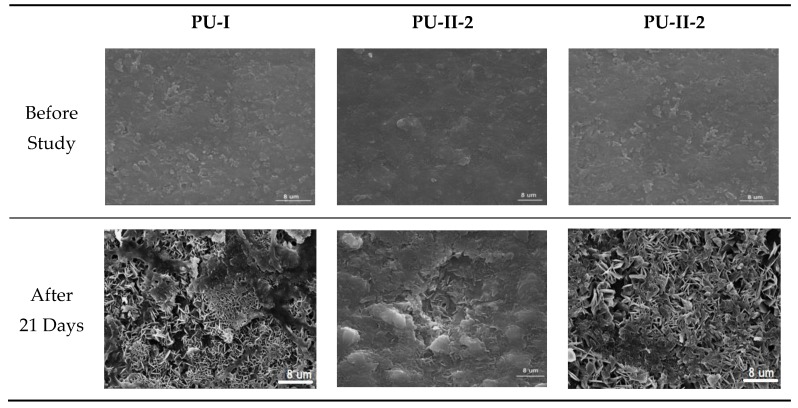
Selected SEM images of non-crosslinked (PU-I) and PVA-crosslinked (PU-II-1, PU-II-2) before and after calcification study.

**Figure 8:**
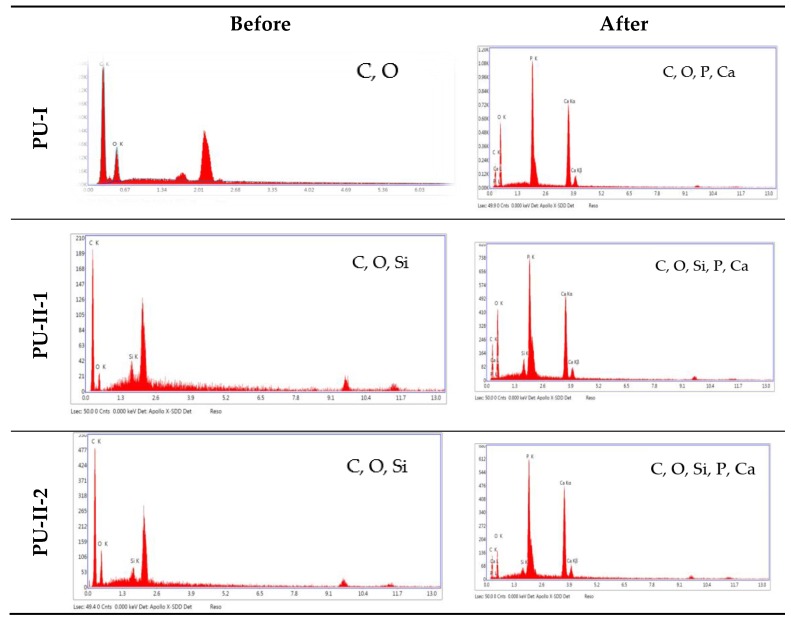
The EDX spectra of non-crosslinked and PVA-crosslinked PURs before and after calcification study.

As can be noted in Figure 7 both non-crosslinked and PVA-crosslinked PURs revealed good ability to calcificy at the surface in properly selected medium. After 21 days of study the obtained material was significantly deposited with calcium salt, which is indicated by the EDX study (Figure 8). 

### 3.8. Hemocompatibility Study

**Figure 9:**
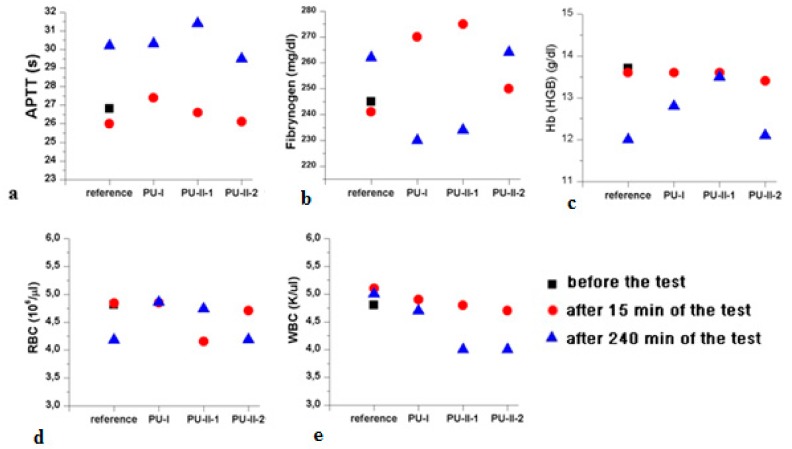
Selected results of PVA-crosslinked and non-crosslinked PURs hemocompatibility. APTT (**a**); Fibrynogen (**b**); Hb (**c**); RBC (**d**); and WBC (**e**).

Figure 9 gathers the selected results of blood parameters tested for non-crosslinked and PVA-crosslinked PURs hemocompatibility. All the studied blood parameters, hemoglobin (Hb), red blood cells, hematocrit (WBC), and the coagulation factors (fibrinogen and partial thromboplastin time APTT), were comparable between PVA-crosslinked PURs and non-crosslinked PUR (Figure 9). It is worth mentioning that the values of the studied blood parameters were in the reference range after 240 min (4 h) of the test. Obtained materials did not significantly influence the blood components.

### 3.9. Biocompatibility

**Figure 10:**
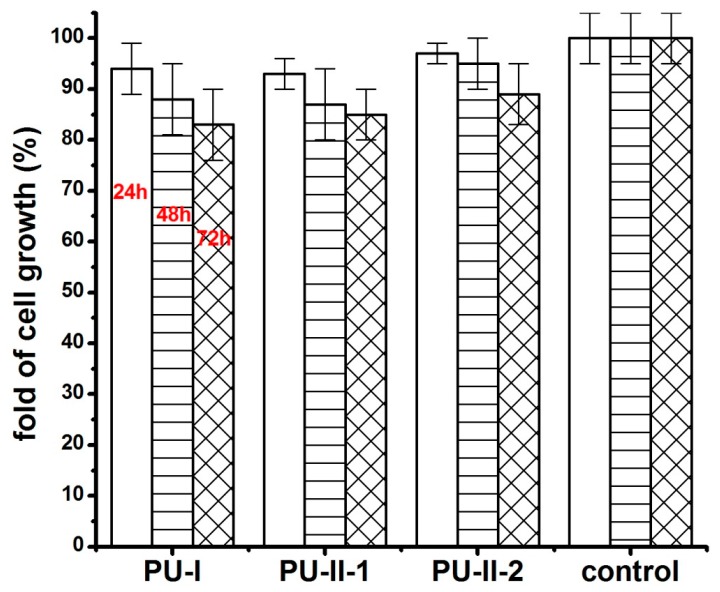
The effect of polymer extracts on the in vitro cell growth of MG 63 studied by MTT assay after 24 h, 48 h and 72 h.

Figure 10 shows the MG 63 cell response on polymer extracts after 24 h, 48 h and 72 h of incubation. It was clearly viewed that both non-crosslinked and PVA-crosslinked PURs possessed good biocompatibility at comparable levels between 83–97% of cell viability. 

**Figure 11:**
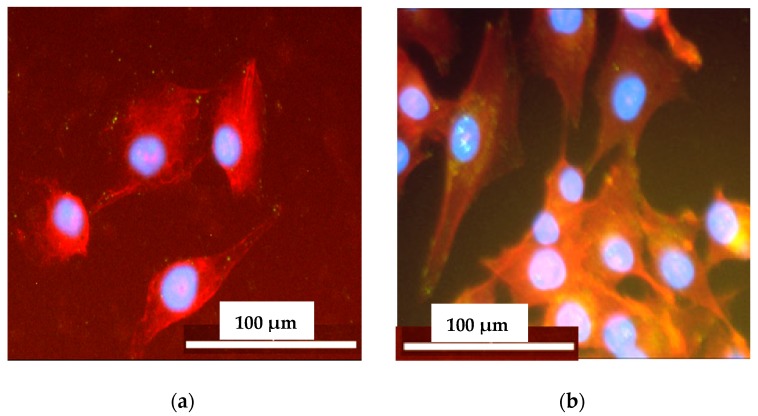
The cell attachment to the selected samples surface: PU-I (**a**) and PU-II-2 (**b**) after 72 h of the test. Cells were simultaneously stained with DAPI (blue) for nucleus visualization and Phalloidin TRITC (red) for actin viability.

After 24 h of culture on non-crosslinked and PVA-crosslinked PURs cells were well attached to the material and spread out, displaying a normal morphology. It indicated that the studied samples (Figure 11) can provide favorable conditions for cell attachment. The elongated and flattened cells adhered to the non-crosslinked and PVA-crosslinked PURs surface.

## 4. Discussion

Tissue engineering is a widely developing field with regard to obtaining functional implants [56,57,58,59]. Crosslinked materials including PUR may play a significant role in in this field [60]. In this paper, was described the concept, synthesis, and characterization of novel, not previously described, PVA-crosslinked PURs as well as the influence of crosslinks on PUR chemical composition, surface characteristics, long-term material behavior in selected media and the material’s biological performance. 

In accordance to performed studies, the increase of PVA in PUR structure (from 1 wt % to 2 wt %) caused the presence of crosslinks, which influenced these materials’ long-term interactions with selected media as well as mechanical properties and hydrophilicity of their surface. Obtained PVA-crosslinked PURs were slowly degradable materials, due to the presence of crosslinks in their structure. In the presence of organic solvents, they were swelling, not dissolving. Sorption of media and solvents by the PUR materials is closely related to their crosslink density. In the case of non-crosslinked PURs selected media more easily penetrated the material in depth and causes sliding of PUR chains, which led to chain defragmentation [10,61]. In comparison, PVA-crosslinked PURs were stable for 3 months of the study in distilled water and PBS. Observed mass decrease did not exceed 5% of the initial weight of the sample. That could be caused by the presence of additional hydrogen bonds formed by crosslinking of PUR by PVA, which added the resistance to the novel materials. It was established that polymer degradation starts through swelling of the medium, which aggregate at the material’s surface and may hinder the medium molecules’ absorption at their surface, which could be related to the additional presence of salts at the material surface [10,61]. Thus, such PVA-crosslinked PURs may be a useful proposition for slowly degradable implants, which may find an application in the field of tissue engineering [59,62] because such materials are able to provide the prolonged physical support of the scaffold until the cells reach a proper level of maturation [63,64,65,66,67]. The surface characteristic of PVA-crosslinked PURs during the long-term interaction study with selected media was not disturbed by occurring infractions or fractures, which is in contrast to the non-crosslinked PURs. The CA of PVA-crosslinked materials was decreased in order to determine the presence of PVA (47° and 38° respectively for PU-II-1 and PU-II-2). In the case of non-crosslinked PURs the CA was higher (59°). It is worth mentioning that the CA of the PVA-crosslinked PURs surface was not significantly changed after 3 months of incubation in PBS. What was interesting was the fact that after 21 days of calcification study, both materials showed a superior ability of calcium deposition. In the long-term interaction profile, CA and calcification study showed that addition of PVA improved hydrophilicity of the obtained materials, whose characteristics were suitable for regenerative medicine purposes [68,69,70]. Liu et al. [69] proved that enhanced surface wettability (contact angle of 75° to 65°) of PURs results in uniform coating with calcium phosphate throughout electrospun scaffold after immersion in simulated body fluid (SBF). It is worth mentioning that Gogolewski and Gorna [18] reported that cancellous bone formed on the scaffolds from PUR with higher content of hydrophilic component contained more bone mineral than the bone formed in the defects implanted with PUR of lower content of hydrophilic component. Moreover, their surface characteristic was suitable for scaffold implantation because the most suitable CA for cell growth at the surface of the implant and their proliferation in depth of mammalian cells is between 45–76° [71,72]. The superior mechanical characteristics of obtained PURs (T_Sb_ of 41 ± 3 MPa and 52 ± 1 MPa for PU-II-1 and PU-II-2 respectively) was comparable to the native bone tissue such as human cortical bone (T_Sb_ in the range of 51–151 MPa) and human cancellous bone (T_Sb_ in the range of 10–20 MPa) [73]. Moreover, the short-term hemocompatibility study showed satisfactory blood-PURs interactions, which is desired in case of medical implants. The slight reduction in the concentration of white blood cells after 4 h of PUR incubation may be related to their natural disintegration and their adhesion to the surface of the PUR materials. Similar observations were made by Paluch et al. [72] who examined the effect of the polyester knitted fabrics, varying in the degree of the wettability, on blood parameters. The authors indicated changes of the blood cells shape by studying them with the use of an electron microscope. It was found that cells had an extended shape and formed aggregates similar to leukocyte-leukocyte and leukocyte-platelet cells. This can indicate the activation of white blood cells, which is the first step of "adaptation" of an implanted artificial material [74,75,76,77]. No exceeding of blood parameter reference values was observed; thus, it can be considered that no inflammation induction was present due to the blood-PUR interaction [74,75,76,77]. The APTT was determined to estimate the coagulation time, which could be disrupted by presence of the foreign body. APTT is the number of seconds required for the formation of the clot (fibrin fibers) in the plasma after exposure to the PUR materials [77]. In the performed study the APTT and fibrinogen were in the reference range, thus the obtained materials did not induce premature blood clotting. This corresponds to the references that relate to the fact that PURs belong to the one of the most hemocompatible synthetic polymers used in the biomedical field [77]. An observed slight increase in erythrocyte (RBC) after 240 min of the test in the case of PU-I compared to the control blood sample can result from an abnormal red blood cell sedimentation caused by the presence of the PUR [77]. PVA-crosslinked PURs did not cause significant changes in RBC value, which was in the reference range of studied blood. Moreover, performed biocompatibility tests with the use of MG 63 cell line revealed that the obtained materials were characterized by great biocompatibility (97–83%) and cell morphology comparable to the control. Thus, the obtained materials may be a suitable candidate for an application in the tissue engineering field.

## 5. Conclusions

In this paper, the design, synthesis, and characterization of novel PVA-crosslinked PURs as a suitable candidate for tissue regeneration are described. The significant changes in terms of swelling properties, surface characteristics and behavior in long-term interactions with selected media were noted. The PVA-crosslinking means that the obtained PUR materials can be classified in the group of slowly degradable materials used in tissue engineering which is suitable in reference to this point. It is worth mentioning that addition of PVA meant that the obtained PURs had hydrophilic surface suitable for cell adhesion and growth. Moreover, novel PVA-crosslinked PURs undergo a uniform calcification within the test. The short-term hemocompatibility study revealed proper blood-PVA-crosslinked PURs interactions, which is suitable for implantation purposes. The great cytocompatibility of the obtained materials was confirmed by cytotoxicity tests with the use of MG 63 cell line. Thus, the obtained PVA-crosslinked PURs may be a suitable candidate for the purposes of regenerative medicine.

## Figures and Tables

**Table 1:** Symbols of obtained PURs and their detailed composition.

Symbol	HDI (g)	PCL (g)	BDO (g)	PVA (g)	The Ratio of NCO:OH in the Prepolymer	Concentration of Free NCO Groups Present in the Prepolymer (%)
PU-I	7.93	27.27	2.87	-	3:1	8.18
PU-II-1	7.93	27.27	-	0.1	3:1	8.18
PU-II-2	7.89	27.11	-	0.2	3:1	8.18

**Table 2:** Band assignments of non-crosslinked and PVA crosslinked PURs.

Wavelength (cm^−1^)	Band Assignment	Description
3350 m	νNH	Stretching of NH groups in urethane bond
1730 vs	νC=O	C=O ester stretching in PCL soft segments
1630 s	νC=O	C=O stretching in urethane hard segments R-NH-COO-R
1570–1540 m	δCN	2º amide N-H urethane deformation
1240 m	νCN	C-N urethane stretching in hard segments
1160 m	ν-(C=O)-O-C-	Combined asymmetric C-O-C stretching in urethane and PCL (Hard/soft segments)
1100, 1080 w	ν-(C=O)-O-C-	Symmetric stretching of C-O-C groups in (1100) PCL/soft and (1080) urethane/hard segments.

vs—very strong, m—medium, w—weak.

**Table 3:** Solubility of obtained PURs in DMSO, the Huggins parameter (χ) and crosslinking density (mol/cm^3^).

Sample	Solubility in DMSO *	χ **	Cross-Links Density (mol/cm^3^)
PU-I	+	−	−
PU-II-1	−	1.1979	31.12 × 10^−4^
PU-II-2	−	1.3579	42.44 × 10^−4^

* + soluble, − swelling; ** Huggins parameter

**Table 4:** Contact angle of obtained PURs before and after 1 and 3 months of incubation in PBS.

Sample	Contact Angle (°)		
	Before	1 Month	3 Months
PU-I	59 ± 0.2	58 ± 0.2	57 ± 0.3
PU-II-1	47 ± 0.1	46 ± 0.1	45 ± 0.2
PU-II-2	38 ± 0.1	37 ± 0.1	37 ± 0.1
